# Regarding “Down but Not Out: Vasectomy Is Faring Poorly Almost Everywhere—We Can Do Better to Make It a True Method Option”

**DOI:** 10.9745/GHSP-D-23-00163

**Published:** 2023-08-28

**Authors:** Michel Labrecque

**Affiliations:** a Faculty of Medicine, Laval University, Quebec City, Canada.

See related articles by Jacobstein et al. and Jacobstein et al.

I salute Jacobstein et al. for their brilliant description of the worldwide state of vasectomy in 2022.^1^ Their analysis and recommendations to increase the use of vasectomy in the family planning mix of methods available globally are comprehensive and clear. The article should be widely disseminated to governmental and nongovernmental funding agencies and organizations.

However, there are limitations associated with using the United Nations (UN) survey database to estimate national prevalence rates for vasectomy and tubectomy. I will use data from Canada as an example. Jacobstein et al. reported that the percentages of vasectomy and tubectomy use in Canadian women of childbearing age were 22.0% and 11.0%, respectively, in 2002 and 7.4% and 7.0%, respectively, in 2006.^1^ Even if no vasectomy had been done in the country over this 4-year period, a relative decrease by 66% (from 22.0% to 7.4%) is unlikely. The vast majority of the couples using vasectomy in 2002 were likely still using this permanent method in 2006. A reference and details about the 2006 survey are not available,^2^ but it does not represent the Canadian reality. Various other sources provide valid estimates of vasectomy use in Canada.

According to the National Physician Database from the Canadian Institute of Health Information, the number of vasectomies and tubectomy were 63,530 and 34,132 (205 and 110 per 100,000 population), 55,488 and 17,662 (161 and 51 per 100,000 population) and 56,545 and 9,450 (150 and 25 per 100,000 population) in 2001–2002, 2011–2012, and 2019–2020, respectively.^3^^,^^4^ The rates per 100,000 population are quite uniform across the Canadian provinces for each period.^3^^,^^4^

We observe that the rate of tubectomy per 100,000 population sharply declined by 77% over these 2 decades. The vasectomy rate also decreased but by much less—a 21% drop between 2001–2002 and 2011–2012. This could not explain the large 66% decline in use of vasectomy reported by the UN between 2002 and 2006.^2^

A shift of some vasectomies from public to private care may explain the apparent small decrease in later years between 2011–2012 and 2019–2020. Before 2017, it was common practice across Canada to bill extra fees to patients for office-based vasectomy. The prohibition of this practice has led to an increase in the number of vasectomies performed by physicians unaffiliated with the universal health care insurance system. With these numbers missing from provincial and national statistics, it is likely that true vasectomy rates are actually increasing. In Quebec province, 14,669 vasectomies in 2021 were billed to the provincial health system (personal communication with the Régie de l’assurance maladie du Québec, email, August 2022), but at least 3,000 of those (17% of the total number) were performed in the private sector.^5^ In British Columbia, this proportion could be as high as 29%, with at least 2,144 vasectomies performed in the private sector in 2019 (personal communication with Jack Chang, MD, from Pollock Clinics, email, April 2023) and 5,129 billed to the provincial health care system during the same period.^4^

The 2014–2015 and 2020–2021 Quebec Population and Health Surveys provided survey results comparable to those reported in the UN database.^6^^,^^7^ The 2014–2015 survey revealed that 18.8% and 7.1% of men aged 15 years and over and 14.7% and 5.3% of women aged 15–49 years were using vasectomy and tubectomy, respectively.^6^ In the 2020–2021 survey, these numbers were very similar for vasectomy (18.6% in men and 14.6% in women) and slightly lower for tubectomy (5.7% in men and 4.9% in women).^7^ In the 2014–2015 survey, vasectomy was the most common contraceptive method used in women aged 35–49 years, with an overall prevalence of 28% and rates as high as 43% in some administrative regions of the province.^8^

As the rate of vasectomy performed per 100,000 population is relatively similar between provinces, the results of the 2014–2015 and 2020–2021 Quebec surveys probably reflect the true prevalence of vasectomy use in Canada. Considering this, it is obvious that the 2006 survey reported in the 2022 UN database largely underestimated prevalence of vasectomy use in Canada.

Although I fully agree with Jacobstein et al.’s conclusions and recommendations, the Canadian example emphasizes the importance of assessing the source data in such a study. Similar flawed estimates may exist in other country data. The validity of original surveys from China and India should have been scrutinized, considering the important contribution of these countries to the overall conclusion of the article. I also question the validity of using surveys performed more than 10 years ago (France [2008], South Korea [2009], Belgium [2010], Bhutan [2010]) for estimating the 2022 prevalence. These limitations of the World Contraceptive Use 2022 data should be acknowledged.

## Notes

**Peer Reviewed**

**First published online:** July 11, 2023.
